# An Overview on the Effect of Neonicotinoid Insecticides on Mammalian Cholinergic Functions through the Activation of Neuronal Nicotinic Acetylcholine Receptors

**DOI:** 10.3390/ijerph17093222

**Published:** 2020-05-06

**Authors:** Jean-Noël Houchat, Alison Cartereau, Anaïs Le Mauff, Emiliane Taillebois, Steeve H. Thany

**Affiliations:** University of Orleans, LBLGC USC INRAE 1328. 1 rue de Chartres, 45060 Orléans, France; jean-noel.houchat@univ-orleans.fr (J.-N.H.); alison.cartereau@univ-orleans.fr (A.C.); anais.le-mauff@univ-orleans.fr (A.L.M.); emiliane.taillebois@univ-orleans.fr (E.T.)

**Keywords:** ACh, cholinergic functions, nicotinic receptors, modulation, neonicotinoid insecticides

## Abstract

Neonicotinoid insecticides are used worldwide and have been demonstrated as toxic to beneficial insects such as honeybees. Their effectiveness is predominantly attributed to their high affinity for insect neuronal nicotinic acetylcholine receptors (nAChRs). Mammalian neuronal nAChRs are of major importance because cholinergic synaptic transmission plays a key role in rapid neurotransmission, learning and memory processes, and neurodegenerative diseases. Because of the low agonist effects of neonicotinoid insecticides on mammalian neuronal nAChRs, it has been suggested that they are relatively safe for mammals, including humans. However, several lines of evidence have demonstrated that neonicotinoid insecticides can modulate cholinergic functions through neuronal nAChRs. Major studies on the influence of neonicotinoid insecticides on cholinergic functions have been conducted using nicotine low-affinity homomeric α7 and high-affinity heteromeric α4β2 receptors, as they are the most abundant in the nervous system. It has been found that the neonicotinoids thiamethoxam and clothianidin can activate the release of dopamine in rat striatum. In some contexts, such as neurodegenerative diseases, they can disturb the neuronal distribution or induce oxidative stress, leading to neurotoxicity. This review highlights recent studies on the mode of action of neonicotinoid insecticides on mammalian neuronal nAChRs and cholinergic functions.

## 1. Introduction

**Figure 1:**
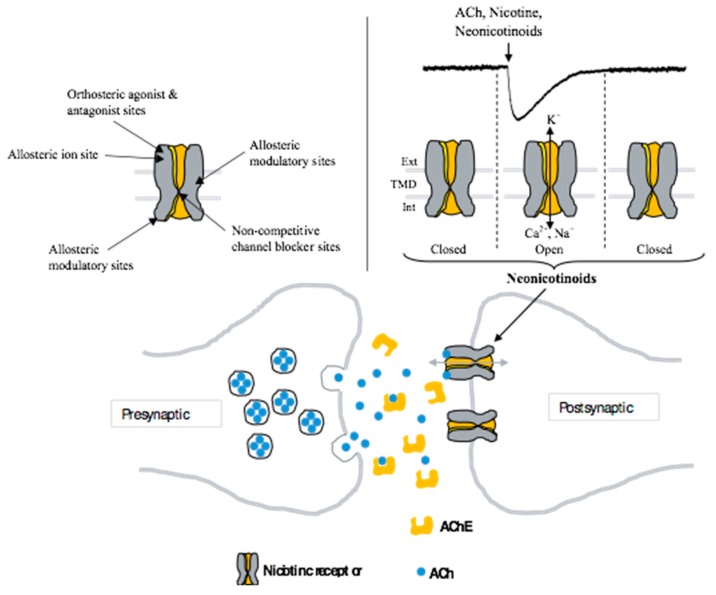
Representation of the cholinergic synapse and ion channel selectivity. The neuronal nicotinic acetylcholine receptors (nAChRs) are located at the postsynaptic terminal. Agonist, antagonist, non-competitive channel blocker, and allosteric sites are represented in the cut-away view showing four of the five subunits forming the pentameric receptor-channel complex. Ext: Extracellular; TMD: transmembrane domain; Int: intracellular.

Neonicotinoid insecticides have contributed to improvements in global agricultural productivity for decades by controlling insects and plant pathogens, providing higher crop yields and improved product quality. Over the last ten years, the extensive use of neonicotinoid insecticides has been criticized due to the risks associated with their toxicity to humans and other non-target species. Neonicotinoid insecticides include several compounds, such as imidacloprid (IMI), acetamiprid (ACE), clothianidin (CLT), and thiamethoxam (TMX), which act on neuronal nicotinic acetylcholine receptors (nAChRs). They are considered as partial or full agonists of insect neuronal nAChRs and poor agonists of mammalian receptors. Full agonists are compounds that, at saturation concentrations, activate the channels to a near 100% open probability. Thus, ACh is a full agonist of nAChRs. Consequently, partial agonists will evoke less total current than full agonists and occupy the same binding site. They can also inhibit receptor activation by endogenous or exogenous full agonists. Thus, the same compound can be a full agonist of one subtype of nAChRs, and a partial agonist or antagonist of another subtype. Moreover, nAChRs are considered as prototypical allosteric proteins that undergo conformational changes upon the binding of a ligand [1,2,3]. Agonists bind to the ligand-binding or orthosteric site on the ligand-binding domain. Allosteric modulators are pharmacological compounds that bind to the receptor at a distinct site from the ligand-binding domain and change the free energy associated with transitions between functional states. This leads to the activation (positive allosteric modulators) or reduction of the ligand-evoked response (negative allosteric modulators) (Figure 1). The cholinergic system in mammals has been studied extensively. It is known that it provides diffuse innervation to the entire brain, driving and modulating a wide variety of behaviors. ACh is released from presynaptic cholinergic axon terminals and binds to the extracellular ligand-binding domain of the nAChRs. Thus, through neuronal nAChRs, ACh regulates processes such as cell excitability and neuronal integration. These processes influence physiological functions, leading to the involvement of neuronal nAChRs in many diseases such as Alzheimer’s and Parkinson’s disease [4,5,6,7]. Given the role of neuronal nAChRs in both insects and mammals, few studies have described the involvement of neonicotinoid insecticides on mammalian cholinergic functions through neuronal nAChRs. Therefore, in this work, we aim to provide an overview of recent studies on the involvement of neonicotinoid insecticides on neuronal cholinergic functions.

## 2. Diversity of Mammalian Neuronal Nicotinic Acetylcholine Receptors

### 2.1. Multiple Receptor Subtypes and Different Pharmacological Properties

Mammalian neuronal nAChRs are of significant importance because cholinergic synaptic transmission plays a key role in rapid neurotransmission, learning, and memory processes. Neuronal nAChRs are composed of 17 homologous genes coding for nAChR subunits [8,9]. These subunits can be divided into two subgroups: the muscle-type receptor composed of a heteromeric receptor (α1βγ(ε)δ), and the neuronal-type receptor, which is more complex in terms of the number of subunits and combinations. They incorporate different subunits and are composed of: (1) Homomeric receptors which are comprised of one subunit, α7, α8, α9, or α10, which can form an α9α10 heteromeric receptor; (2) heteromeric receptors, which are formed by subunits α2–α6 in combination with β2–β4. Consequently, multiple receptor subtypes can be formed with different stoichiometry, subunit combinations, and pharmacological properties [10]. Thus, when opened, neuronal nAChRs are cation-selective channels that permit the flow of sodium (Na^+^), potassium (K^+^), and calcium (Ca^2+^) ions across the membrane (Figure 1). A net influx of cations through the channel pore depolarizes the cell membrane and increases neuronal excitability. The impact of neuronal nAChR activation on cholinergic function depends on subunit composition because each nAChR subtype has unique activation, agonist selectivity, channel conductance, and desensitization properties. The vast majority of subunit combinations have not been studied, and a significant number of publications on neonicotinoid modes of action refer to the low-affinity homomeric α7 and high-affinity heteromeric α4β2 receptors because they are the most abundant in the nervous system [11]. For heteromeric receptors such as α4β2, the ratio of subunits forming the receptors may have an influence on the pharmacological properties of the receptors [12,13,14,15,16,17]. For example, receptors with the composition (α4β2)_2_α4 exhibit higher single-channel conductance and a shorter mean open lifetime than receptors with the composition (α4β2)_2_β2 [16]. Moreover, long-term exposure of α4β2 receptors to nicotine causes an increase in the number of binding sites at the cell surface, known as up-regulation, whereas α7 desensitizes rapidly in response to high agonist concentrations [18] and has a high relative permeability to calcium [19,20]. Indeed, it is known that nicotine has a low affinity for α7 receptors, and high doses of nicotine may be required for the regulation of the α7 receptor. The different modulatory effect of nicotine on both α7 and α4β2 nAChRs suggests that different mechanisms may activate the up-regulation of α7 and α4β2.

### 2.2. Neonicotinoids Are Poor Activators of Mammalian Neuronal nAChRs

The discovery of neonicotinoid insecticides from a lead compound, leading to nithiazine, then followed by the synthesis of IMI, has been described in various degrees [21,22,23]. Nicotine and neonicotinoids have some structural similarities (especially IMI), and act on the same receptor subtype but in a different way. Interactions between neuronal nAChRs and neonicotinoids are studied by measuring inward currents caused by neonicotinoids. It has been recognized that neonicotinoids activate insect neuronal nAChRs as agonists in different ways [24]. As one of the most widely used neonicotinoid insecticides in the world, IMI is known as a partial agonist, while CLT and ACE are “super” agonists. In contrast, TMX is a poor agonist, despite being able to activate synaptic activity [25]. Unfortunately, there are few studies relating to the mode of action of neonicotinoid insecticides on mammalian neuronal nAChRs. Using clonal rat pheochromocytoma (PC12) cells, Nagata et al. demonstrated that IMI weakly activated nAChRs with conductance states identical to those of ACh-generated currents [26]. Moreover, using a single-channel patch-clamp method, they found that co-application of both IMI and ACh resulted in a decrease in the mean open time and mean burst duration of the currents of main conductance states, compared with those induced by ACh alone [26]. Ihara et al. found that IMI, nitenpyram (NTP), and CH-IMI (the nitromethylene analog of IMI), all induced inward currents which were rapidly desensitized. However, IMI and NTP were partial agonists on the α7, whereas CH-IMI and DN-IMI (the desnitro derivative of IMI) were full agonists because their currents were closed to that of the ACh [27]. Similarly, on the α4β2 receptors, it was found that IMI, CH-IMI, and NTP were inactive as agonists, whereas DN-IMI—which lacks the nitro group—was a full agonist [27]. The weak action of neonicotinoids on the α4β2 receptor was attributed in part to the β2 subunit lacking basic residues in loop D. This led to the idea that “insect-selective” residues confer neonicotinoid sensitivity by direct interactions or changes in the α subunit conformations [27]. Overall, this poor agonist action of neonicotinoids on α7 and α4β2 nAChRs explains the lack of substantial data concerning the effect of these compounds on other mammalian nAChRs, considering their diversity within the mammalian nervous system.

## 3. Detoxification Mechanisms in Mammals in Regards to Neonicotinoid Sensitivity

The higher sensitivity of insects to neonicotinoid insecticides relative to mammals may be due to several factors. Firstly, it may relate to enzymatic detoxification mechanisms, which are important considering that the metabolite will not affect the receptor target. In this case, it has been considered that changes in xenobiotic-metabolic enzyme expression, and particularly the overexpression of cytochrome P450, are associated with increased neonicotinoid resistance in insects [28,29,30,31,32,33,34]. Thus, the toxicity of neonicotinoids in insects could be explained as a lack of, or decrease in, the physiological activity of detoxifying enzymes [35,36]. This difference was found between the bees *Apis mellifera* and *Apis cerana,* in which it was demonstrated that *A. cerana* was more sensitive to IMI and CLT compared to *A. mellifera*, in part because glutathione-S-transferase activity was significantly higher in *A. mellifera* [36]. Unfortunately, no study refers to the effectiveness of these enzymatic mechanisms in mammals with regards to neonicotinoid resistance or sensitivity. Considerable efforts have been made to identify neonicotinoid insecticide metabolism in mammals [37,38,39,40,41,42,43]. Human CYP450 enzymes, and in particular, CYP3A4, 2C19, and 2B6, have been found to convert TMX to CLT. CYP3A4, 2C19, and 2A6 metabolized CLT to desmethy l-CLT, and CYP2C19 converted TMX to desmethyl-TMX [42]. These enzymes were involved with considerable amounts of neonicotinoid substrates, which increased or decreased in different parts of the mammalian body, such as the liver and brain. In other studies, it was considered that because of this enzymatic activity, neonicotinoids such as TMX could be hepatotoxic and hepatocarcinogenic [44,45,46]. Consequently, the complex activity of detoxifying enzymes in mammals is no longer associated with their ability to provide resistance to neonicotinoids, but with their capacity to increase toxicity. It was also proposed that insecticides are more effective at the ambient temperature of insects (around 15−20 °C) than that of mammals (if we consider 36 °C as a reference temperature). However, this hypothesis seems to have been challenged by findings that neonicotinoids show significantly lower efficacy at low temperatures (between 14 and 22 °C) when used to manage the *Drosophila suzukii (Matsumura)* [47]. Acute toxic assays on aquatic insects such as the mayfly *Isonychia bicolor* demonstrated an increase in IMI uptake with increasing environmental temperatures [48]. According to these studies, it appears that an increase in environmental temperature was more effective as a factor inducing physiological variations, leading to neonicotinoid toxicity in the insects. Indeed, Mao et al. proposed that the sensitivity of *Nilaparvata lugens* to NTP and other insecticides increased significantly when the temperature changed from 18 to 36 °C. They also found that this increase in sensitivity was correlated to a decrease in cytochrome P450 activity [49]. Their latter observation appears more comprehensive considering that a great proportion of mammals, including humans, have a temperature around 36 °C, and that a decrease in detoxifying enzyme activities will result in the activation of the neonicotinoid targets. Thirdly, if we agree that most neonicotinoids undergo metabolic modifications at multiple sites in both insects and mammals, we must presume that the mechanisms by which neonicotinoids could be toxic to mammals are predominantly associated with their neuronal targets, the nAChR subtypes. Thus, two hypotheses can be made: (i) neonicotinoids will directly activate neuronal nAChRs as agonists, leading to excitation of the cholinergic system; (ii) neonicotinoids are not able to activate (or poorly activate) mammalian neuronal nAChRs at a binding site and will be considered as modulators. Thus, much of the remaining knowledge concerning the toxic effect of neonicotinoids should be considered in regard to their modulatory activity on mammalian cholinergic function and neuronal nAChRs.

## 4. Alterations of Cholinergic Functions

### 4.1. Modulation of Mammalian Neuronal nAChR Function

**Figure 2:**
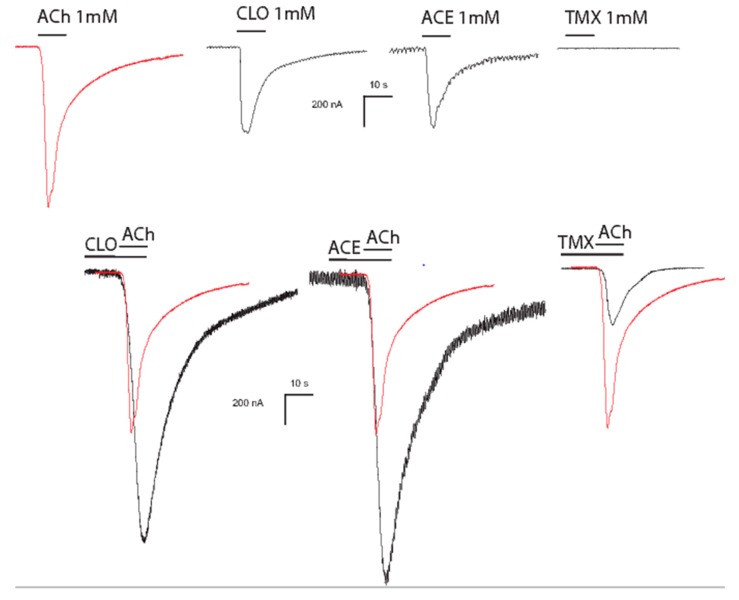
Effects of neonicotinoids on ACh-evoked current amplitudes. Clothianidin (CLT) and acetamiprid (ACE) enhance ACh-induced current amplitudes of the mammalian α7 neuronal nAChRs and thiamethoxam (TMX) decreases ACh-induced current amplitudes.

The major problem found with neonicotinoid insecticides is to always consider them as agonists of neuronal nAChRs (Figure 2). Indeed, despite neonicotinoids being poor activators of neuronal nAChRs, several studies have demonstrated that they can interact with nAChR agonists [50,51,52]. In a previous study, Matsuda et al. found that the responses of α4β2 to ACh were potentiated by IMI [53]. Toshima et al. proposed that ACh-evoked currents through chicken α4β2 receptors can be potentiated by CLT and IMI [52]. To address the mechanism of potentiation, they studied the effect that co-application of IMI and CLT had on the concentration-response curve of ACh. In the presence of IMI and CLT, the ACh concentration–response curve for α4β2 was shifted to the left, whereas thiacloprid (THC) shifted the curve to the right, and was also able to inhibit ACh-evoked currents [52]. These results demonstrated that IMI, CLT, and THC have differential action on mammalian neuronal nAChRs, which may be due to their activity on a particular site in the nAChRs [54]. Thus, we propose that the first effect of neonicotinoids on mammalian neuronal nAChRs is to disrupt nAChR responses to the endogenous ligand, ACh. Exposure of HEK cells expressing human α4β2 receptors to CLT and IMI showed inward currents of low amplitudes. However, IMI strongly reduced ACh responses, whereas CLT enhanced the responses. This difference was associated with the subunit stoichiometry of α4β2 receptors containing three α subunits rather than two α subunits (as with IMI), and CLT inhibited ACh-evoked currents [50]. One of the major questions has been to demonstrate whether a similar effect would be seen with a homomeric receptor where the fifth position is occupied by the same subunit. Recently, using α7 homomeric nAChRs, we demonstrated that the co-application of low concentrations of CLT and acetamiprid (ACE) with ACh did not change ACh-evoked current amplitudes. However, pretreatment before the application of ACh significantly increased ACh-evoked currents by almost two-fold [51]. To confirm the modulatory effect of neonicotinoids, we used TMX, which is known as ineffective against mammalian neuronal nAChRs. A low concentration of TMX decreased the ACh-induced currents through the α7 receptor when it was co-applied or pretreated [51]. All these results reinforce the idea that the mode of action of neonicotinoid insecticides is more attributable to their modulatory effect on mammalian receptors than agonist efficacy. Indeed, through the modulatory effect we are able to understand why they can disturb cholinergic synaptic transmission.

### 4.2. Are Neonicotinoid Insecticides Able to Interact with Mechanisms Involved in Neurodegenerative Diseases?

The involvement of pesticides in neurodegenerative diseases has been the subject of several polemics, particularly over the past 10 years. This is due to the extensive use of pesticides in the environment and growing evidence demonstrating that they can disturb the development of the mammalian central nervous system [55,56,57]. Controversies have also been fueled by the fact that epidemiological evidence is far from conclusive, as considerable heterogeneity has been observed between the patients and chemicals involved. In addition, neurodegenerative diseases are complex syndromes resulting from different genetic and environmental factors that give rise to various degrees of cognitive deficits, motor deficits, and other functions. Nevertheless, oxidative stress and apoptosis have been well investigated as neurotoxic mechanisms leading to the toxic effect of several pesticides [58,59,60]. However, few studies have indicated that neonicotinoid exposure could be associated with neurodegenerative disease. Recently, Dhouib et al. suggested that curcumin, which has anti-inflammatory, antioxidant, and anti-tumor properties, protects rats against ACE-induced cerebellum toxicity, such as an increase in AChE activities, a decline in cell viability, and oxidative stress [61]. Moreover, Kagawa and Nagao found that mice embryos exposed to ACE from day 6 to day 13 developed hypoplasia of the cortical plate and decreased neurogenesis. Newborn ACE-exposed mice showed an abnormal neuronal distribution in the neocortex, increased numbers of the microglial marker Iba1, and the active microglia had a globular structure (amoeboid-type microglia) [57]. In some contexts, in pathological states such as Parkinson’s disease, it has been proposed that inappropriate microglial activation contributes to neurodegeneration through the production of cellular oxidants and cytokines [62,63]. The current treatment for Alzheimer’s disease (AD) is acetylcholinesterase (AChE) inhibitors, which partially block the degradation of ACh in the synapse and enable more of the neurotransmitter to reach and activate cholinergic receptors [64]. It was also found that nAChR density decreased with disease progression, suggesting a link between beta-amyloid (Aβ) and nAChR function [65]. Soluble Aβ species, particularly oligomeric Aβ1−42, interacts with several nAChR subtypes [66]. In addition, AD is also associated with the deterioration of memory and cognitive function. With a base chemical structure similar to nicotine, and because neuronal nAChRs are considered to be affected in several neurodegenerative diseases [65,66], additional studies are needed to further explore the potential involvement of neonicotinoid insecticides on neurodegenerative diseases through neuronal nAChRs.

### 4.3. Neonicotinoid Insecticides Affect Other Mammalian Neuronal Mechanisms

Mammalian neuronal nAChRs are involved in several functions, such as the release of dopamine in the striatal region, glutamatergic synapse formation, and brain development [67,68,69]. The effect of IMI on the properties of stellate cells of the ventral cochlear nucleus (VCN) demonstrated that it increased neuronal excitability and caused a depolarizing shift in the membrane potential. The IMI effect was blocked by the specific nicotinic receptor antagonists d-tubocurarine (d-TC) and α-bungarotoxin (α-Bgt). The blocking of α-Bgt suggested that α7 homomeric receptors were involved in the spontaneous action potential induced by IMI [56]. The modulatory effect of neonicotinoids on mammalian nervous systems was also studied through the activation of catecholamine release. It was found that *in vivo*, TMX and its metabolite CLT induced the release of dopamine in the rat striatum. The CLT activated α4β2 and α7 to induce an *in vivo* striatal release of dopamine. Intrastriatal infusion of CLT increased extracellular dopamine levels, which was blocked by pretreatment with nAChR antagonists, N-n-decylnicotinium iodide (NDNI), dihydro-β-erythroidine (DHβE), and methyllycaconitine (MLA) [70]. Moreover, it was found that IMI facilitated tyrosine hydroxylase (TH) transcription via the activation of α3β4 neuronal nAChRs and α7 receptors. Activation took place at concentrations that are known to produce physiological responses such as catecholamine secretion through the nAChRs in adrenal chromaffin cells. Thus, it was proposed that IMI facilitated the physiological functions of adrenal glands in mammals [71]. In other studies, it was found that exposure to TMX altered behavioral and biochemical processes related to the cholinergic systems in rats. Acetylcholinesterase (AChE) activity was measured in different brain regions such as the hippocampus, striatum, and cortex. It was found that TMX reduced spontaneous motor activity and decreased AChE activity in the hippocampus, cortex, and striatum. The inhibition of AChE activity was long-standing and was accompanied by deficits in behavioral performance [72]. Although the mechanisms leading to the effect of TMX on these brain structures are not well understood, it was hypothesized that TMX activates neuronal nAChRs, leading to an increase in serotonin release, which could explain the anxiogenic effect observed in rats during the plus-maze test [72]. Moreover, all these studies raise the question of a link between neonicotinoids and the blood–brain barrier (BBB) penetration. Indeed, no studies have highlighted a direct adverse effect of neonicotinoids on the BBB, though some have demonstrated an increase of BBB permeability after pyrethrinoid uptake [73,74]. The penetration of neonicotinoids into the mammalian central nervous system has been attributed to their hydrophobicity, which is greater than nicotine [75]. Considering their potential toxic effect through the activation of neuronal nAChRs, studies on the toxicological capacity of neonicotinoids to alter the BBB mechanisms are critical.

## 5. Conclusions

In summary, the enzymatic activity of CYP450 and other enzymes was not sufficient to demonstrate that they can activate resistance in mammals in the same way as in insects. The most evident finding is that neuronal nAChRs are major factors involved in neonicotinoid toxicity in mammalian central nervous systems. We suggest here that neonicotinoid insecticides could differently activate or modulate each neuronal nAChR. The main mechanisms in mammals seem to be a modulatory effect, which will have a consequence on the activation of receptors and the modulation of synaptic activity. Moreover, mammals express different nAChR subtypes in the peripheral and central nervous systems. The modulatory effect of neonicotinoids found with α7 and α4β2 suggests that similar mechanisms need to be explored in other mammalian neuronal nAChRs.
